# Systematic review and meta-analysis of the association between Epstein–Barr virus, multiple sclerosis and other risk factors

**DOI:** 10.1177/1352458520907901

**Published:** 2020-03-23

**Authors:** Benjamin M Jacobs, Gavin Giovannoni, Jack Cuzick, Ruth Dobson

**Affiliations:** Preventive Neurology Unit, Wolfson Institute of Preventive Medicine, Queen Mary University of London, London, UK; Preventive Neurology Unit, Wolfson Institute of Preventive Medicine, Queen Mary University, London, UK/Blizard Institute, Queen Mary University of London, London, UK/Royal London Hospital, London, UK; Preventive Neurology Unit, Wolfson Institute of Preventive Medicine, Queen Mary University of London, London, UK; Preventive Neurology Unit, Wolfson Institute of Preventive Medicine, Queen Mary University of London, London, UK/Royal London Hospital, London, UK

**Keywords:** Multiple sclerosis, clinically isolated syndrome, Epstein–Barr virus, infectious mononucleosis, systematic review, meta-analysis

## Abstract

**Background:**

Epstein–Barr virus (EBV) infection is thought to play a central role in the development of multiple sclerosis (MS). If causal, it represents a target for interventions to reduce MS risk.

**Objective:**

To examine the evidence for interaction between EBV and other risk factors, and explore mechanisms via which EBV infection may influence MS risk.

**Methods:**

Pubmed was searched using the terms ‘multiple sclerosis’ AND ‘Epstein Barr virus’, ‘multiple sclerosis’ AND EBV, ‘clinically isolated syndrome’ AND ‘Epstein Barr virus’ and ‘clinically isolated syndrome’ AND EBV. All abstracts were reviewed for possible inclusion.

**Results:**

A total of 262 full-text papers were reviewed. There was evidence of interaction on the additive scale between anti-EBV antibody titre and HLA genotype (attributable proportion due to interaction (AP) = 0.48, *p* < 1 × 10^−4^). Previous infectious mononucleosis (IM) was associated with increased odds ratio (OR) of MS in HLA-DRB1*1501 positive but not HLA-DRB1*1501 negative persons. Smoking was associated with a greater risk of MS in those with high anti-EBV antibodies (OR = 2.76) but not low anti-EBV antibodies (OR = 1.16). No interaction between EBV and risk factors was found on a multiplicative scale.

**Conclusion:**

EBV appears to interact with at least some established MS risk factors. The mechanism via which EBV influences MS risk remains unknown.

## Introduction

Multiple sclerosis (MS) is thought to arise as the result of acquired environmental risk in a genetically susceptible population.^1–4^ Environmental risk factors for MS include Epstein–Barr virus (EBV) infection, smoking, obesity during adolescence, and low serum vitamin D.^2^ Understanding how environmental risk factors interact with each other and with genotype is crucial to developing targeted preventive strategies.

We set out to update and extend our understanding of the interaction between EBV and other MS risk factors. To our knowledge, there has been no previous attempt to integrate all data related to how EBV interacts with other MS risk factors. One meta-analysis has examined the potential interaction between EBV serostatus and human leukocyte antigen (HLA) in MS; other previous meta-analyses have not studied risk factor interaction.^5–8^

Interaction can be defined as the situation in which the relationship between exposure and outcome depends, in some way, on the presence or value of some other exposure. It is important to distinguish between biological interaction – the claim that there are physical, mechanistic relationships between the exposures – and statistical interaction – a directly estimable property from observed data on the probability of the outcome given different combinations of exposures. Inferring biological interaction from statistical interaction is not trivial and requires additional mechanistic evidence to show biological plausibility.

Studying interaction(s) in the pathogenesis of MS is important for several reasons: it can identify individuals in whom specific exposures are of particular importance, which has implications for who to target with prevention studies, and it sheds light on disease pathogenesis by identifying overlapping causal pathways to disease. For instance, the observation that obesity interacts with HLA genotype suggests not only that anti-obesity measures are particularly important in individuals with high-risk HLA haplotypes, but also argues for the effect of obesity on MS risk being immune-mediated.

Statistical interaction can be conceived of on two scales: additive interaction (or ‘departure from additivity’), where the risk of the outcome exceeds the sum of risk conferred by each exposure, or multiplicative interaction, where the risk of the outcome exceeds the product of the relative risks for each exposure. For public health purposes, for example, deciding which subgroups of individuals will benefit more from a treatment or vaccine, additive interaction is the more relevant measure as it captures absolute benefit (i.e. total number of diseases prevented), which can be missed on the multiplicative scale if baseline risks in the two groups are different.^9^

Nested case-control studies using large health repositories^10,11^ have made a major contribution to epidemiological evidence supporting a causal relationship between EBV and MS. However, the high rate of EBV seropositivity in the general population argues against EBV seropositivity alone being a sufficient factor for causing MS.^7^ The prevalence of MS in EBV-negative individuals is virtually zero when highly sensitive techniques are used to assess EBV serostatus.^12,13^ Symptomatic EBV^5–7,14–16^ infection (infectious mononucleosis (IM)) confers a greater risk of MS than asymptomatic EBV carriage.

Population-based epidemiological studies indicate that EBV infection and other environmental risk factors may interact with genotype in the pathogenesis of MS.^17^ To our knowledge, there have been no previous attempts to systematically pool these estimates. In this systematic review and meta-analysis, we examine all the available evidence for EBV interaction with other MS risk factors (in terms of both EBV serostatus and IM) using both multiplicative and additive models for interaction. We also examine the reported relationship between EBV and MS, and pool evidence around the relationship between active EBV turnover (as measured by polymerase chain reaction (PCR)) and MS. Finally, we provide a narrative systematic review of the literature around MS and EBV.

## Methods

### Search strategy

Pubmed was searched using the terms ‘multiple sclerosis’ AND ‘Epstein Barr virus’, ‘multiple sclerosis’ AND EBV, ‘clinically isolated syndrome’ AND ‘Epstein Barr virus’ and ‘clinically isolated syndrome’ AND EBV. Search dates were 1950–present. The most recent search was performed on 22 December 2018.

All abstracts were reviewed for possible inclusion. Studies for use in the meta-analysis were screened according to the following criteria: containing both MS and control group, and using either standard techniques to establish EBV serostatus, history of IM or PCR. Where these criteria were met, the full text was retrieved.

Following this, relevant studies were reviewed and data extracted. Where full text was not available, the authors were contacted to provide the article. Where it was judged unclear as to whether data within selected papers met the inclusion criteria (details of inclusion criteria for each analysis are given in the ‘Results’ section), a second co-author independently reviewed the paper, and a consensus decision was reached. The quality of data was assessed by recording the reported security of MS diagnosis (no clear criteria and/or self-reported vs explicit criteria used for diagnosis, the gold standard), and technique for assessing EBV (enzyme-linked immunosorbent assay (ELISA) vs immunofluorescence, the gold standard). All references of retrieved review and/or meta-analyses were reviewed for additional articles not captured during the original search.

Technical differences between study design may introduce bias and limit the validity of pooled effect estimates. Such differences included differences in clinical criteria for MS diagnosis, differences in method of IM diagnosis (clinical, recall questionnaire, serological), differences in laboratory techniques (e.g. immunofluorescence vs ELISA), differences in HLA genotyping (molecular typing vs single-nucleotide polymorphism (SNP) imputation), and difference in the quantification of smoking exposure (cotinine vs questionnaire). To overcome these difficulties, we performed subgroup analyses where appropriate to stratify by these potential sources of heterogeneity (e.g. by method of HLA genotyping).

All included full-text papers were assigned to analyses covered by this review – EBV interaction with other MS risk factors, serology and MS risk, IM and MS risk, EBV DNA detection and MS and papers covering possible mechanisms of EBV contribution to MS. A single paper could be assigned to any number of analyses, and each analysis/review was performed independently of all others.

### Statistical methods

Meta-analyses were conducted in R v3.6.1 using the ‘meta’ package based on reported data. Odds ratios (ORs) were calculated using a Mantel–Haenszel random-effects model with a continuity correction. Bias was quantified using the efficient score (a linear regression of funnel plot asymmetry).^18^ For interaction studies, ORs were pooled using inverse variance-weighted meta-analysis. Where data were available, unadjusted ORs were calculated.

For interaction studies, the highest and lowest exposure groups were used – for example, where Epstein–Barr Virus Nuclear Antigen (EBNA) titres were divided into quartiles, we took the lowest and the highest groups as ‘EBNA lo’ and ‘EBNA hi’ respectively. Interaction was assessed by calculating four measures of interaction: where the numbers of cases and controls in each risk factor group were presented, the attributable proportion due to interaction (AP), the relative excess risk due to interaction (RERI), the synergy index (S), and multiplicative interaction^19,20^ were calculated. For two risk factors of interest, for example, smoking and HLA status, OR_11_ indicates the OR for MS in individuals exposed to both risk factors, OR_10_ the OR for HLA + non-smokers and OR_01_ that for HLA-smokers

$$\mathrm{RERI}={\mathrm{OR}}_{11}-{\mathrm{OR}}_{10}-{\mathrm{OR}}_{01}+\;1$$

$$S=\frac{{\mathrm{OR}}_{11}-1}{\left(\left({\mathrm{OR}}_{10}-1\right)-\left({\mathrm{OR}}_{01}-1\right)\right)}$$

$$\mathrm{AP}=\frac{\left({\mathrm{OR}}_{11}-{\mathrm{OR}}_{10}-{\mathrm{OR}}_{01}+\;1\right)}{{\mathrm{OR}}_{11}}$$

In the absence of interaction, RERI and AP will be 0, and S will be 1. Measures of departure from additivity (AP, RERI and S) were calculated using the indicator variable method described previously^20^ in R v 3.6.1. As standard errors can only be computed for the natural log of the synergy index, we have presented this measure as log(synergy index) ± 95% confidence intervals (CIs). A null effect (no interaction) would give a log(synergy index) of 0 (ln(1) = 0). Multiplicative interaction was calculated by performing logistic regression with an interaction term. If OR represents the OR for MS, x_1_ one risk factor, x_2_ the second risk factor, and x_1_x_2_ the product (interaction) term, then

$$\mathrm{Ln}\left(\mathrm{OR}\right)=b_{0}{+b}_{1}x_{1}{+b}_{2}x_{2}{+b}_{3}x_{1}x_{2}$$

The exponent of the interaction term coefficient b_3_ represents the multiplicative interaction between the two risk factors. For these analyses, the regression model did not adjust for variables other than the two risk factors in question. Standard errors were calculated for measures of additive interaction using the delta method.^19^ Standard errors for the multiplicative interaction were calculated from the output of the logistic regression model. Meta-analysis of interaction terms was performed using the inverse variance method with a random-effects model.

### Data and code availability statement

This work was performed using published data. All data sources are listed in the references and supplementary references. All R code used for the analysis is available on Github (https://github.com/benjacobs123456/EBV_meta_analysis/blob/master/analysis.R).

## Results

**Figure 1.**
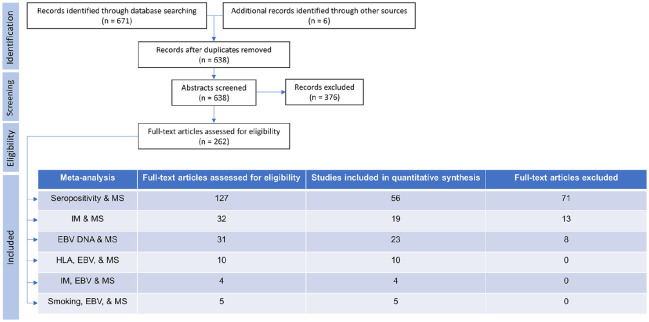
PRISMA flow charts with details of publications retrieved via searches, abstracts screened, full-text articles assessed and used in analyses.

A total of 632 references were retrieved using the search terms ‘multiple sclerosis’ AND ‘Epstein Barr virus’, and ‘multiple sclerosis’ AND EBV. ‘Clinically isolated syndrome’ AND ‘Epstein Barr virus’ retrieved 22 references, all of which had been captured in the previous search. ‘Clinically isolated syndrome’ AND EBV retrieved a further 17 references, again all of which had been previously captured. Review of all references of meta-analyses and systematic reviews provided six unique new results. A total of 370 results were discarded following review of abstracts for reasons such as pre-selecting EBV positive patients only, having no control group, and validation studies of new methods for EBV serology. A total of 262 full-text papers were reviewed and included as summarised in Figure 1.

### EBNA titre interaction with HLA-DRB1*1501 in MS

Ten papers^21–29^ were included for this analysis. All but one paper presented HLA-DRB1*1501 homo- and heterozygotes pooled into a single group (‘HLA positive’), and so this grouping was used in the analysis. Where EBNA titres were divided into quartiles, we took the highest and lower quartiles to represent ‘high’ and ‘low’ titres, respectively. One paper^27^ was excluded due to overlapping participants with another paper.^23^

**Figure 2.**
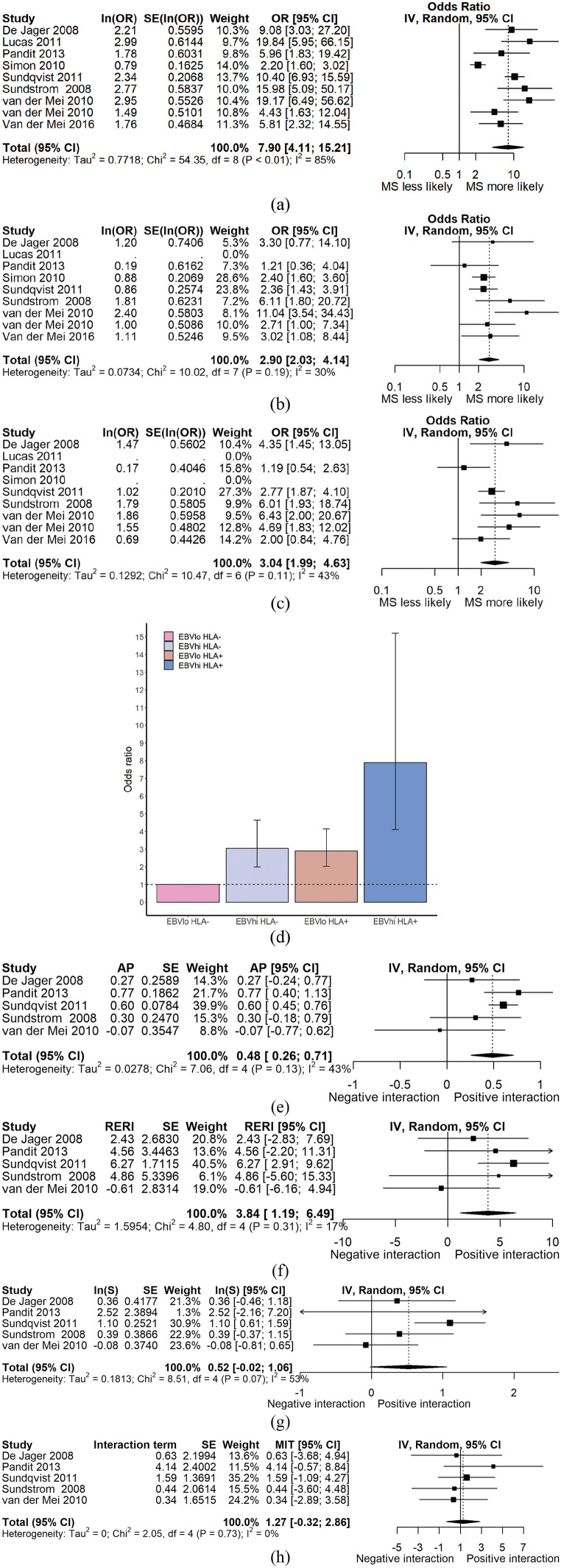
(a) Forest plot demonstrating OR_MS_ for HLA^+^EBNA^hi^ persons. (b) Forest plot demonstrating OR_MS_ for HLA^+^EBNA^lo^ persons. (c) Forest plot demonstrating OR_MS_ for HLA^–^EBNA^hi^ persons. (d) Bar chart demonstrating evidence of interaction between HLA-DRB1*1501 genotype and EBNA antibody titre on an additive, but not multiplicative scale. The dotted line represents the null (OR = 1). (e)–(h) Forest plots demonstrating estimates of interaction – AP, RERI, synergy index, and multiplicative interaction respectively – for studies with individual-level data available. The reference group (with OR = 1) is HLA^–^EBNA^lo^ individuals for all panels. MIT: multiplicative interaction term.

The OR of MS in individuals with high anti-EBV antibody titres is increased in HLA-DRB1*1501 positive (OR = 7.90, 95% CI = 4.11–15.21) compared to HLA-DRB1*1501 negative individuals (OR = 3.04, 95% CI = 1.99–4.63, Figure 2, Table 1). Studies differed in their method of HLA genotyping. Restricting the analysis to studies using tagging SNPs (rs3135005 or rs9271366) did not significantly alter the results (Supplemental Figure S1). Restricting the analysis to studies using PCR-based methods yielded a similar result (Supplemental Figure S1).

**Table 1.** Odds ratios and 95% CIs for MS in each stratum of EBNA titre and HLA genotype. In the top half of the table, odds ratios are derived from meta-analysis of all studies. In the bottom half, estimates of additive and multiplicative interaction are shown with their standard errors. These estimates are derived from only those studies with individual-level data (i.e. number of participants in each stratum) available.

	HLA^–^	HLA^+^
EBNA lo (OR; 95% CI)	1 (reference)	2.90 (2.03–4.14)
EBNA hi (OR; 95% CI)	3.04 (1.99–4.63)	7.90 (4.11–15.21)
	Estimate	*SE* (*p*)
AP	0.49	0.12 (3.09E – 05)
RERI	3.84	1.35 (0.004)
Log(synergy index)	0.52	0.28 (0.059)
Multiplicative interaction	1.27	0.81 (0.739)

EBNA: Epstein–Barr Virus Nuclear Antigen; OR: odds ratio; CI: confidence interval; AP: attributable proportion due to interaction; RERI: relative excess risk due to interaction.

Individual-level data were available for five studies. We estimated the degree of interaction between HLA status and EBNA titre by calculating the AP, synergy index, RERI, and the degree of multiplicative interaction as described above. There was evidence of significant interaction between EBNA titre and HLA genotype on the additive scale in terms of the AP and RERI (AP = 0.48, *p* < 1 × 10^−4^; RERI = 3.84, *p* < 5 × 10^−3^; S = 1.68, *p* = 0.06). There was no evidence of interaction on the multiplicative scale (β = 1.27, *p* = 0.74) (Figure 2, Table 1). Subgroup analyses based on method of HLA genotyping are presented in Supplemental Table S1.

### IM interaction with HLA-DRB1*1501 in MS

**Figure 3.**
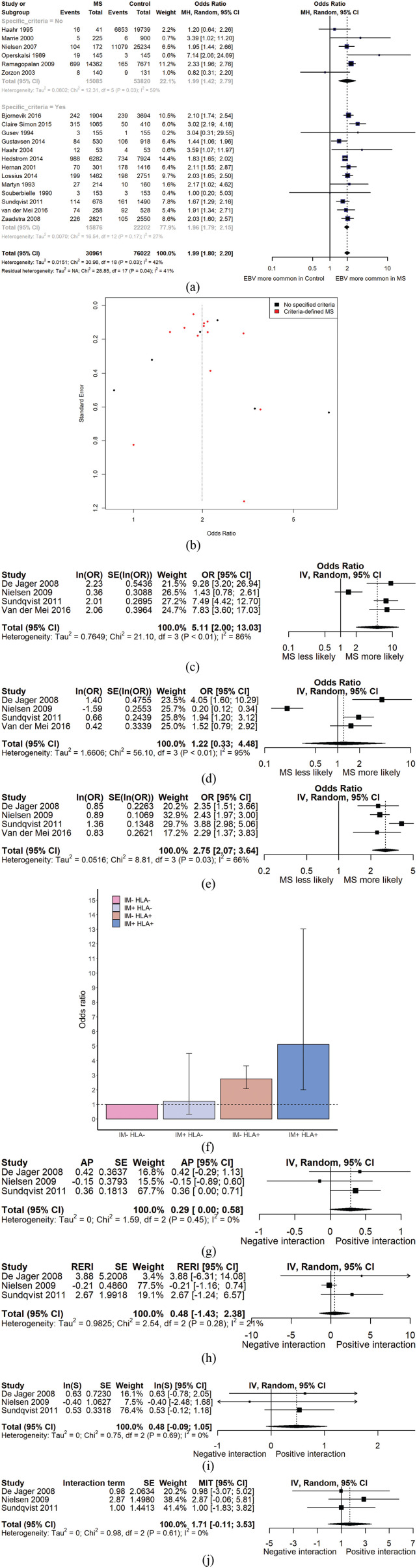
(a) Forest plot of studies examining the relationship between previous infectious mononucleosis and MS. (b) Funnel plot demonstrating no clear evidence of publication bias in these studies. (c) Forest plot demonstrating OR_MS_ for HLA^+^IM^+^ persons. (d) Forest plot demonstrating OR_MS_ for HLA^–^IM^+^ persons. (e) Forest plot demonstrating OR_MS_ for HLA^+^IM^–^ persons. (f) Bar chart demonstrating lack of evidence of interaction between HLA-DRB1*1501 genotype and prior IM on an additive, but not multiplicative scale. The dotted line represents the null (OR = 1). (g)–(j) Forest plots demonstrating estimates of interaction – AP, RERI, synergy index, and multiplicative interaction respectively – for studies with individual-level data available. The reference group (with OR = 1) is HLA^–^IM^–^ individuals for all panels. MIT: multiplicative interaction term.

To estimate the prevalence of prior IM among controls and people with MS, we reviewed 32 full-text papers, of which 19 met the inclusion criteria (supplementary references). Inclusion criteria were MS and control group, clearly stated methods for obtaining a previous history of IM, and no selection on the basis of reported history of IM. Previous IM was more common in people with MS (OR = 2.00, 95% CI = 1.80–2.20, *p* < 0.0001, Figure 3). There was significant heterogeneity (Q = 31.0, *p* = 0.03) but no evidence of publication bias (*p* = 0.62, Figure 3). This effect persisted after restricting studies to those using criteria-defined MS (OR = 1.94, 95% CI = 1.81–2.07, Figure 3).

Four papers examined the potential interaction between previous IM and HLA-DRB1*1501 status and MS,^21,23,29.30^ Again, homo- and heterozygote status was pooled into ‘HLA positive’. A history of IM is associated with increased OR of MS in HLA-DRB1*1501 positive individuals (OR = 5.11, 95% CI = 2.00–13.03; *p* < 1 × 10^−3^) but not in HLA-DRB1*1501 negative individuals (OR = 1.22, 95% CI = 0.33–4.48; *p* = 0.77, Figure 3, Table 2). Three studies had individual-level data available. There was no significant interaction on the additive or multiplicative scales between HLA status and IM in the meta-analysis of three studies with individual-level data available (Figure 3, Table 2). Subgroup analysis by method of HLA genotyping did not significantly alter the results (Supplemental Figure S2).

**Table 2.** Odds ratios and 95% CIs for MS in each stratum of IM and HLA genotype. In the top half of the table, odds ratios are derived from meta-analysis of all studies. In the bottom half, estimates of additive and multiplicative interaction are shown with their standard errors. These estimates are derived from only those studies with individual-level data (i.e. number of participants in each stratum) available.

	HLA^–^	HLA^+^
IM^–^ (OR; 95% CI)	1 (reference)	2.75 (2.07–3.64)
IM^+^ (OR; 95% CI)	1.22 (0.33–4.48)	5.11 (2.00–13.03)
	Estimate	*SE* (*p*)
AP	0.29	0.15 (0.053)
RERI	0.48	0.97 (0.624)
Log(synergy index)	0.48	0.29 (0.100)
Multiplicative interaction	1.71	0.93 (0.443)

OR: odds ratio; CI: confidence interval; AP: attributable proportion due to interaction; RERI: relative excess risk due to interaction.

### EBV interaction with smoking in MS

**Figure 4.**
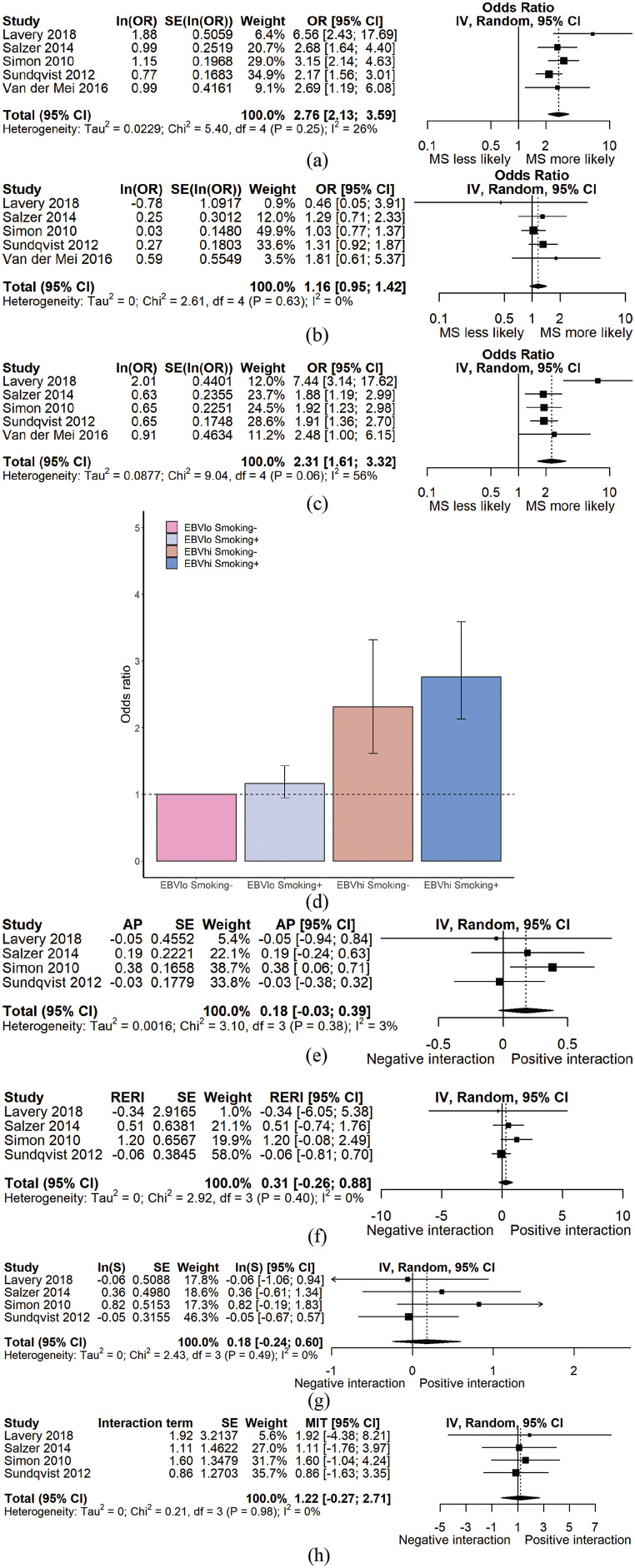
(a) Forest plot demonstrating OR_MS_ for EBNA^hi^Smoking^+^ persons. (b) Forest plot demonstrating OR_MS_ for EBNA^lo^Smoking^+^ persons. (c) Forest plot demonstrating OR_MS_ for EBNA^hi^Smoking^–^ persons. (d) Bar chart demonstrating lack of evidence of interaction between smoking status and EBNA titre on an additive, but not multiplicative scale. (e)–(h) Forest plots demonstrating estimates of interaction – AP, RERI, synergy index, and multiplicative interaction respectively – for studies with individual-level data available. The reference group (with OR = 1) is EBNA^lo^Smoking^–^ individuals for all panels. MIT: multiplicative interaction term.

Five papers studied the potential interaction between smoking status and anti-EBV antibody titre.^26,27,29,31,32^ Three studies stratified smoke exposure as ever vs never smokers, one study used second-hand smoke exposure as a variable, and one study distinguished active from inactive smoking using serum cotinine levels. Smoking is associated with a greater risk of MS in those with high anti-EBV antibodies (OR = 2.76, 95% CI = 2.13–3.59; *p* < 1 × 10^–5^) but not in those with low anti-EBV antibodies (OR = 1.16, 95% CI = 0.95–1.42; *p* = 0.15). There was no significant interaction on the multiplicative or additive scales in the meta-analysis of the four eligible studies (Figure 4, Table 3). Exclusion of either the study using second-hand smoke as the exposure or using serum cotinine as a proxy for smoking did not significantly affect the results (Supplemental Table S2).

**Table 3.** Odds ratios and 95% CIs for MS in each stratum of EBNA titre and smoking status. In the top half of the table, odds ratios are derived from meta-analysis of all studies. In the bottom half, estimates of additive and multiplicative interaction are shown with their standard errors. These estimates are derived from only those studies with individual-level data (i.e. number of participants in each stratum) available.

	Smoking^–^	Smoking^+^
EBNA lo (OR; 95% CI)	1 (reference)	1.16 (0.95–1.42)
EBNA hi (OR; 95% CI)	2.31 (1.61–3.32)	2.76 (2.13–3.59)
	Estimate	*SE* (*p*)
AP	0.19	0.13 (0.125)
RERI	0.42	0.47 (0.348)
Log(synergy index)	0.22	0.21 (0.280)
Multiplicative interaction	1.38	0.79 (0.629)

OR: odds ratio; CI: confidence interval; AP: attributable proportion due to interaction; RERI: relative excess risk due to interaction.

### EBV interaction with vitamin D in MS risk

Only two studies presented data on both EBV and vitamin D in MS.^33,34^ One of these studies looked at vitamin D levels in people with established MS,^33^ and the other in samples taken both prior to and following MS onset, with multiple, variable sampling points per participant.^34^ One study applied a correction to vitamin D levels for month of sampling,^34^ the other did not.^33^ In addition, one study used a single EBNA epitope,^34^ whereas the other looked at specific EBNA-1 domains.^33^ Neither study demonstrated any interaction between vitamin D level and anti-EBNA titre; however, for the reasons above, they were not pooled.

### EBV interaction with obesity in MS risk

Only one study examined the potential interaction between EBV and obesity in risk of MS.^35^ This study demonstrated a striking potential interaction on an additive scale with an AP of 0.8 (95% CI = 0.6–1.0) in the incident study, and in the prevalent study an AP of 0.7 (95% CI = 0.5–1.0).^35^

### EBV seropositivity and MS

Fifty-six papers were included in the final analysis for this analysis (supplementary references). Inclusion criteria were MS and control group, no pre-selection of groups based on EBV serostatus and history of IM, EBV serology measured using clearly defined methods. Reasons for exclusion included not having a control group and pre-selecting EBV positive patients. Studies were separated into those examining adult vs paediatric MS populations given the reported differences in seroprevalence between the two groups. Following an assessment of data quality, validatory analyses were performed, limiting studies to those deemed to be of high quality. Seropositivity for EBV was calculated by pooling results from studies which reported seropositivity to either EBNA, viral capsid antigen (VCA), or both. Where both were reported, the EBNA data were used. Studies using different EBNA1 and EBNA2 epitopes were pooled for all analysis.

**Figure 5.**
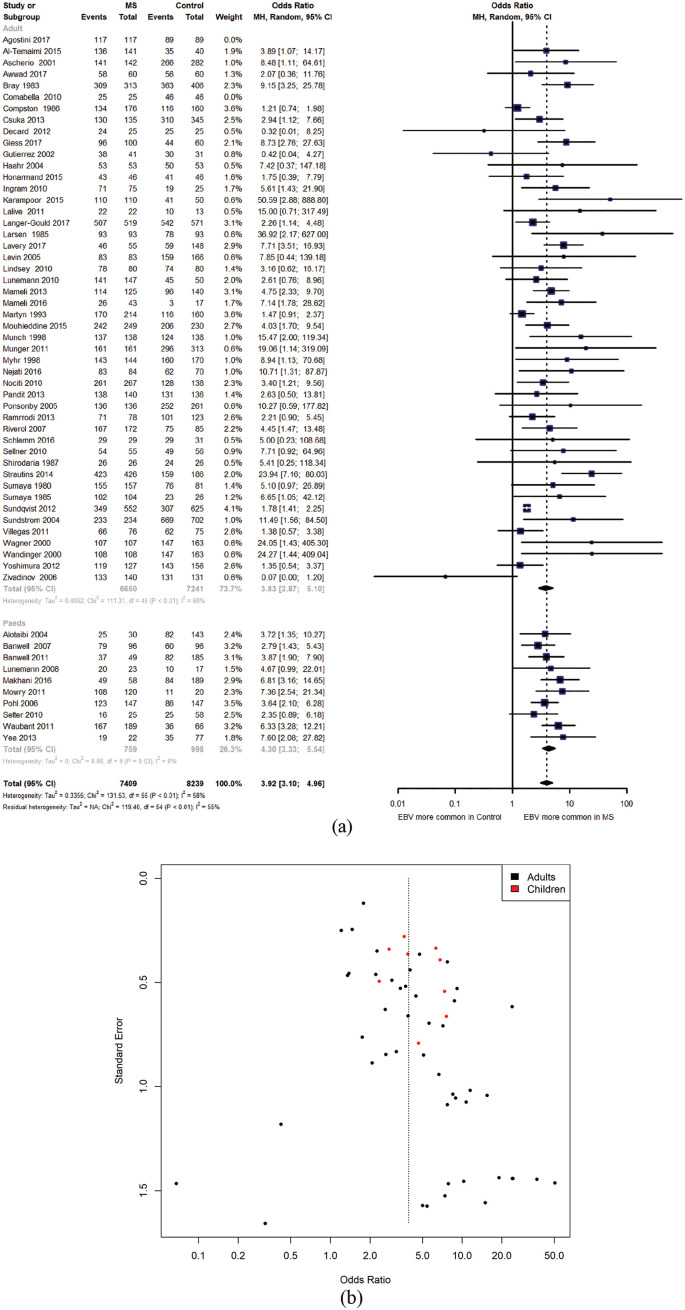
(a) Combined forest plot with meta-analysis of EBV seropositivity in children and adults with MS. Odds ratios represent the odds ratio for EBV seropositivity given a diagnosis of MS (i.e. odds of EBV seropositivity among people with MS/odds of EBV seropositivity among controls). (b) Funnel plot demonstrating evidence of publication bias in publications examining EBV seropositivity and MS.

EBV seropositivity was significantly more common among people with MS (adults and children) than controls (OR_(EBV seropositivity|MS status)_ = 3.92, 95% CI = 3.10–4.96, *p* < 0.0001, Figure 5). There was evidence of significant heterogeneity (Q = 131.53, *p* < 1 × 10^−4^) and publication bias (*p* < 0.05). Overall, 6868/7459 people with MS were EBV seropositive (92.1%) compared with 6231/8266 EBV seropositive control subjects (81.4%).

EBV seropositivity was more prevalent among adults with MS compared to controls (OR_(EBV seropositivity|MS status)_ = 3.83, 95% CI = 2.87–5.10, *p* < 0.0001). There was substantial heterogeneity between studies (Q = 111.3 *p* < 1 × 10^−4^) and evidence of publication bias (*p* = 0.012), with studies demonstrating a relationship between EBV infection and MS more likely to be published. Overall, 6225/6700 adults with MS were EBV seropositive (92.9%) compared with 6220/7268 adult control subjects (85.6%). EBV seropositivity was more common among children with MS or clinically isolated syndrome (CIS) than controls (OR_(EBV seropositivity|MS status)_ = 4.30, 95% CI = 3.33–5.54, *p* < 0.0001). There was no evidence of heterogeneity (Q = 8.1, *p* = 0.52) and no evidence of publication bias (*p* = 0.75). Overall, 643/759 children with MS were EBV seropositive (84.7%) compared with 511/998 control subjects (51.2%).

IgG reactivity to VCA was more prevalent among adults with MS (OR = 3.23, 95% CI = 2.05–5.10, *p* < 1 × 10^−4^, data not shown).

There was substantial heterogeneity between studies (Q = 53.3, *p* = 0.0002) and no evidence of publication bias (*p* = 0.12). Reactivity to the EBNA antigen was again more prevalent among people with MS compared to controls (OR = 3.63, 95% CI = 2.69–4.89, *p* < 1 × 10^−4^, data not shown). There was substantial heterogeneity between studies (Q = 73.2, *p* < 1 × 10^−4^) with evidence of publication bias in these studies (*p* < 0.003).

The increased seroprevalence of EBV infection in people with MS/CIS remained significant when restricting included studies to those using the more sensitive technique of immunofluorescence (rather than ELISA) to detect EBV antibodies (OR = 4.62, 95% CI = 2.24–9.53). Similarly, when restricting included studies to those which used explicit diagnostic criteria to define MS, this effect remained significant (OR = 3.47, 95% CI = 2.64–4.56).

### EBV DNA detectable by PCR

A total of 31 full-text papers were reviewed and 23 included in the analysis (supplementary references). Eight papers studied EBV DNA in cerebrospinal fluid (CSF), three in whole blood, seven in peripheral blood mononuclear cells, four in plasma/serum and one in saliva. The EBNA gene was the most commonly used for EBV detection (nine studies), with Bam used in four studies, VCA in three studies, and latent membrane protein (LMP) in two studies.

EBV DNA was detectable in whole blood/PBMC more often in people with MS versus controls (*n* = 1853, nine studies, OR = 3.48, 95% CI = 1.7360–6.9659, *p* < 5 × 10^−4^). There was evidence of significant heterogeneity (Q = 48.94, *p* < 1 × 10^−4^) but no evidence of publication bias (*p* = 0.78). Detection of EBV DNA did not differ between MS and control serum/plasma samples (*n* = 607, OR = 1.81, 95% CI = 0.77–4.26, *p* = 0.18) or CSF (*n* = 802, OR = 1.74, 95% CI = 0.97–3.12, *p* = 0.062).

### Discussion and conclusions

There is a considerable body of epidemiological evidence implicating EBV in the pathogenesis of MS. EBV infection appears to be a necessary but not sufficient requirement for developing MS, EBV seroprevalence is higher among people with MS, symptomatic EBV infection (IM) is more prevalent among people with MS, and HLA-DRB1*1501 genotype modifies the effect of anti-EBV antibody titre on MS risk.

In our meta-analysis of interaction between EBV and other risk factors, we demonstrate evidence for supra-additive interaction between EBNA titre and HLA status in determining risk. The absence of strong evidence for interaction between EBV and other risk factors in our analysis demonstrates the importance of using multiple measures of interaction (AP, RERI, synergy index and multiplicative interaction) to avoid the risk of type 1 error. However, the small number of studies suitable for our analysis of interaction and the presence of substantial heterogeneity between studies limits the power of this meta-analysis, and therefore, conclusions about interaction should be drawn cautiously from these results.

We observed significant heterogeneity in the HLA-EBNA and HLA-IM analyses. Although we overcome some of this heterogeneity by using random-effects meta-analysis, we acknowledge that this heterogeneity not only questions the validity of combining such studies, but also is a likely source of imprecision that may bias the estimates of interaction. Sources of such heterogeneity include differences in EBNA antigen and detection method, different EBNA titre distributions within studies, different methods of HLA genotyping, different distributions of HLA alleles within the populations studies, different methods of IM diagnosis, and other differences between the populations studied such as age, gender split and exposure to other risk factors which may confound the associations. We have attempted to reduce the heterogeneity in these estimates by performing various pre-specified sensitivity analyses (e.g. by method of HLA genotyping). Reassuringly, these sensitivity analyses aligned with the primary analyses. Nonetheless, we emphasise that our results alone should not be overinterpreted due to the substantial heterogeneity between studies.

Another important limitation of our study is that, in order to calculate standard errors for measures of additive interaction (AP, RERI and synergy index), raw data are required regarding the number of participants in each stratum of exposure. To adjust for confounding, the number of participants in each stratum of the confounder must also be known. As these data are not publicly available, our estimates of interaction are calculated without adjustment for confounding, which clearly has the potential to bias the study-level and meta-analysed estimates of interaction. It is possible to calculate measures of interaction (but not their standard errors) from the output of multivariate logistic regression models (which are adjusted for confounding): although the number of included studies was greater in these analyses (Tables 1–3), these estimates did not differ dramatically from the measures of interaction calculated from studies with raw data available (RERI HLA-EBNA: 1.94; RERI HLA-IM: 2.14; RERI Smoking-EBNA: 0.29). These results suggest that our analyses have limited power to detect a true interaction, but do not suggest that our results are biased.

The mechanism via which EBV exerts this increased risk remains unknown, and our systematic review of the literature highlights a multitude of potential biological mechanisms that have been both demonstrated, replicated, and importantly not replicated. It seems likely that the route via which EBV exerts its effect lies in complex interactions between EBV and the host genome, the precise mechanisms of which remain to be elucidated.

Large prospective cohort and case-control studies have provided strong evidence implicating IM in the pathogenesis of MS.^18^ Although formal analysis of interaction did not reveal interaction between IM and HLA, the OR for MS differed strikingly between IM^+^HLA^–^ individuals (OR = 1.22) and IM^+^HLA^+^individuals (OR = 5.11). These observations suggest that IM may be a more significant predictor of MS risk in HLA DRB1*1501 carriers. Practically, this hypothesis would have important implications for targeted MS prevention, as it would suggest that IM prevention (e.g. with an EBV vaccine)^2^ should be targeted to DRB1*1501 carriers to maximise benefit. Our data alone do not provide a sufficiently strong case for this strategy, but do add to the argument that this approach may be effective.

Our results for the seroprevalence of EBV among people with MS are consistent with the previously published meta-analysis, which reported ORs of 4.47 (95% CI = 3.26–6.11) and 4.51 (95% CI = 2.84–7.16) for EBNA and VCA, respectively. Our estimates of 3.63 (95% CI = 2.69–4.89) and 3.23 (95% CI = 2.05–5.10) are more conservative, likely reflecting new, larger studies with smaller effect sizes and our different inclusion criteria.^7^ Similarly, our estimates of measures of interaction between EBNA titre, HLA status, and MS risk are similar, though not identical, to the published meta-analysis estimates.^8^ The previous study used fixed-effects meta-analysis as opposed to random-effects (which we use here) to pool estimates of interaction, but other reasons for this discrepancy are not clear.

Despite the evidence above, not all epidemiological aspects of MS can be explained by EBV infection. The relatively short latency between putative infection and subsequent MS seen in the Faroe epidemics, and the decreasing risk in migrants moving from high- to low-risk areas cannot be explained purely by EBV infection – the fact remains that MS is overwhelmingly likely to be the result of multiple environmental risk modifiers. However, evidence for EBV infection as an obligate step in MS development is increasing and, with vaccination on the horizon as a potential preventive intervention, cannot be ignored.

## Supplemental Material

MSJ907901_supplemental_material – Supplemental material for Systematic review and meta-analysis of the association between Epstein–Barr virus, multiple sclerosis and other risk factors Click here for additional data file. Supplemental material, MSJ907901_supplemental_material for Systematic review and meta-analysis of the association between Epstein–Barr virus, multiple sclerosis and other risk factors by Benjamin M Jacobs, Gavin Giovannoni, Jack Cuzick and Ruth Dobson in Multiple Sclerosis Journal
