# Supplementary material for: Systematic review and meta-analysis of the association between Epstein–Barr virus, multiple sclerosis and other risk factors
Source: Mult Scler. 2020 Mar 23;26(11):1281–97. doi: 10.1177/1352458520907901 (PMC7543008; doi:10.1177/1352458520907901)
Supplement: MSJ907901_supplemental_material – Supplemental material for Systematic review and meta-analysis of the association between Epstein–Barr virus, multiple sclerosis and other risk factors [file MSJ907901_supplemental_material.pdf]

Table S1: interaction effect estimates for interaction between HLA status and EBNA titre – subgroup analysis based on method of HLA genotyping.

PCR-based HLA genotyping			
	Estimate	SE	P.value
AP	0.600626	0.080903	1.14E-13
RERI	5.848862	1.473361	7.20E-05
Log(Synergy index)	0.8520740	0.29822258	4.274305e-03
Multiplicative interaction	1.771796	1.030103	0.453711
Tag SNP HLA genotyping			
	Estimate	SE	P.value
AP	0.148602	0.209132	0.477353
RERI	0.988962	1.947507	0.611587
Log(Synergy index)	0.1139513	0.2786410	0.6825735
Multiplicative interaction	0.446705	1.320622	0.675241

Table S2: interaction effect estimates for interaction between smoking status and EBNA titre – subgroup analysis with one study excluded due to second-hand smoke exposure being used as the exposure, and one for using serum cotinine as a proxy measure for smoking.

Excluding study assessing second-hand smoke			
	Estimate	SE	P.value
AP	0.188255	0.127964	0.14125
RERI	0.39963	0.372714	0.283623
Log(Synergy index)	0.2422447	0.2503367	0.3332064
Multiplicative interaction	1.179697	0.781378	0.818112
Excluding study using cotinine as proxy for smoking			
	Estimate	SE	P.value
AP	0.159765	0.158914	0.314724
RERI	0.377317	0.477298	0.429221
Log(Synergy index)	0.1489028	0.2585146	0.5646194
Multiplicative interaction	1.263166	0.888417	0.767063

Supplementary figure legends

Supplementary figure 1: (a)-(d) Graphs as per figure 2, analysis restricted to studies using tagging SNPs to determine HLA genotype. (e) - (h) Graphs as per figure 2, analysis restricted to studies using PCR-based methods to determine HLA genotype.

Study	ln(OR)	SE(ln(OR))	Weight	OR [95% CI]
De Jager 2008	2.21	0.5595	14.1%	9.08 [3.03; 27.20]
Lucas 2011	2.99	0.6144	13.1%	19.84 [5.95; 66.15]
Simon_NHS 2010	1.55	0.8790	9.2%	4.70 [0.84; 26.32]
Simon_Tas 2010	0.79	0.3275	18.6%	2.20 [1.16; 4.18]
van der Mei 2010	2.95	0.5526	14.2%	19.17 [6.49; 56.62]
van der Mei 2010	1.49	0.5101	15.0%	4.43 [1.63; 12.04]
Van der Mei 2016	1.76	0.4684	15.8%	5.81 [2.32; 14.55]
**Total (95% CI)**			**100.0%**	**6.77 [3.43; 13.37]**

Heterogeneity: $Tau^2 = 0.5421$; $Chi^2 = 18.32$, df = 6 ($P < 0.01$); $I^2 = 67\%$

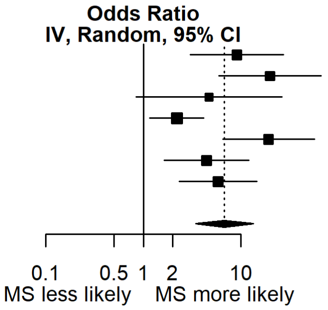

(a)

Study	ln(OR)	SE(ln(OR))	Weight	OR [95% CI]
De Jager 2008	1.20	0.7406	12.4%	3.30 [0.77; 14.10]
Lucas 2011	.	.	0.0%	
Simon_NHS 2010	0.47	0.1339	29.6%	1.60 [1.23; 2.08]
Simon_Tas 2010	1.59	1.2141	6.1%	4.90 [0.45; 52.92]
van der Mei 2010	2.40	0.5803	16.1%	11.04 [3.54; 34.43]
van der Mei 2010	1.00	0.5086	18.1%	2.71 [1.00; 7.34]
Van der Mei 2016	1.11	0.5246	17.7%	3.02 [1.08; 8.44]
**Total (95% CI)**			**100.0%**	**3.15 [1.63; 6.07]**

Heterogeneity: $Tau^2 = 0.3614$; $Chi^2 = 13.28$, df = 5 ($P = 0.02$); $I^2 = 62\%$

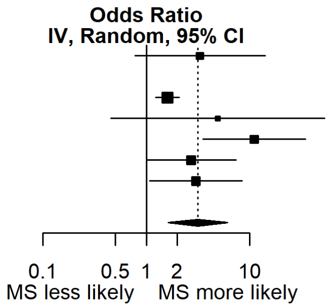

(b)

Study	ln(OR)	SE(ln(OR))	Weight	OR [95% CI]
De Jager 2008	1.47	0.5602	20.8%	4.35 [1.45; 13.05]
Lucas 2011	.	.	0.0%	
Simon_NHS 2010	.	.	0.0%	
Simon_Tas 2010	.	.	0.0%	
van der Mei 2010	1.86	0.5958	18.5%	6.43 [2.00; 20.67]
van der Mei 2010	1.55	0.4802	28.0%	4.69 [1.83; 12.02]
Van der Mei 2016	0.69	0.4426	32.7%	2.00 [0.84; 4.76]
**Total (95% CI)**			**100.0%**	**3.70 [2.23; 6.16]**

Heterogeneity: $Tau^2 = 0.0106$; $Chi^2 = 3.12$, df = 3 ($P = 0.37$); $I^2 = 4\%$

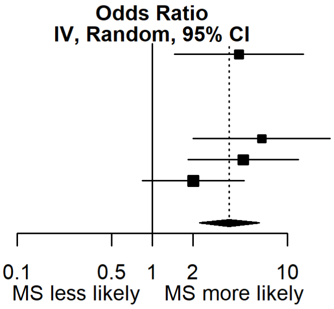

(c)

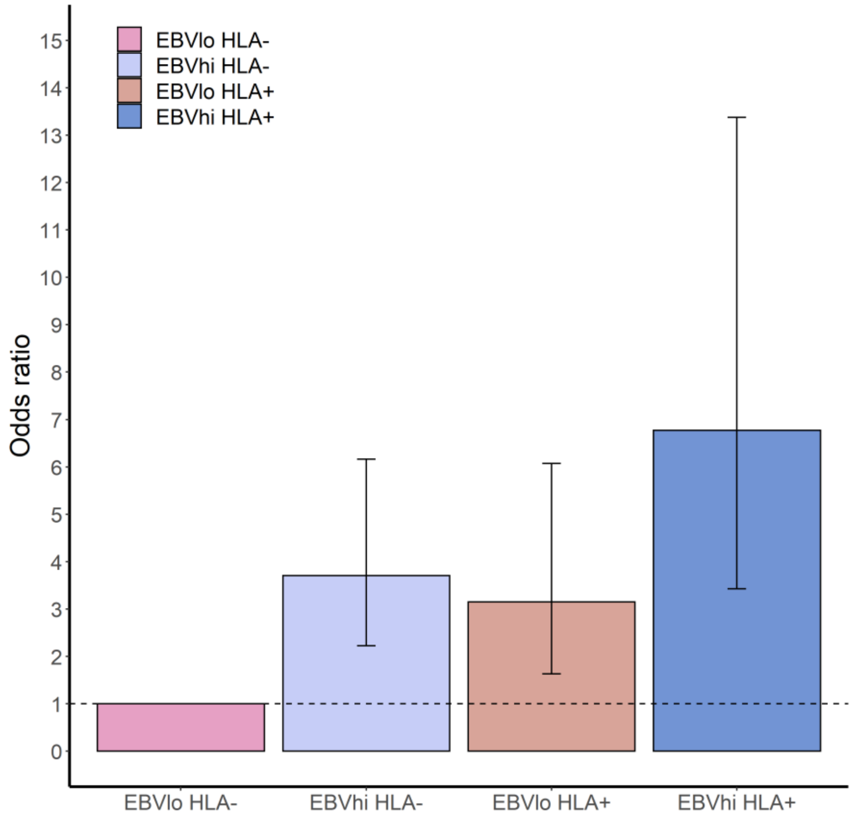

(d)

Study	ln(OR)	SE(ln(OR))	Weight	OR [95% CI]
Pandit 2013	1.78	0.6031	16.1%	5.96 [1.83; 19.42]
Simon_Swedish 2010	0.96	0.6675	14.7%	2.60 [0.70; 9.62]
Sundqvist 2011	2.34	0.2068	25.9%	10.40 [6.93; 15.59]
Sundqvist 2012	1.20	0.1683	26.6%	3.31 [2.38; 4.60]
Sundstrom 2008	2.77	0.5837	16.6%	15.98 [5.09; 50.17]
**Total (95% CI)**			**100.0%**	**6.13 [2.97; 12.68]**

Heterogeneity: $Tau^2 = 0.4868$; $Chi^2 = 23.17$, df = 4 (P < 0.01); $I^2 = 83\%$

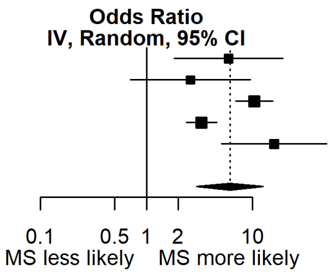

(e)

Study	ln(OR)	SE(ln(OR))	Weight	OR [95% CI]
Pandit 2013	0.19	0.6162	5.8%	1.21 [0.36; 4.04]
Simon_Swedish 2010	1.34	0.8003	3.4%	3.80 [0.79; 18.24]
Sundqvist 2011	0.86	0.2574	31.0%	2.36 [1.43; 3.91]
Sundqvist 2012	1.10	0.1886	54.2%	3.01 [2.08; 4.36]
Sundstrom 2008	1.81	0.6231	5.6%	6.11 [1.80; 20.72]
**Total (95% CI)**			**100.0%**	**2.78 [2.08; 3.72]**

Heterogeneity: $Tau^2 = 0.0054$; $Chi^2 = 4.16$, df = 4 (P = 0.39); $I^2 = 4\%$

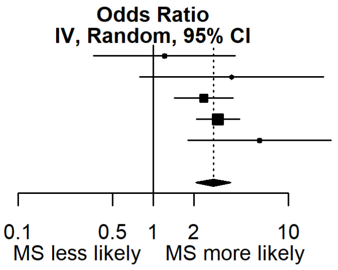

(f)

Study	ln(OR)	SE(ln(OR))	Weight	OR [95% CI]
Pandit 2013	0.17	0.4046	32.1%	1.19 [0.54; 2.63]
Simon_Swedish 2010	.	.	0.0%	
Sundqvist 2011	1.02	0.2010	44.9%	2.77 [1.87; 4.10]
Sundqvist 2012	.	.	0.0%	
Sundstrom 2008	1.79	0.5805	23.0%	6.01 [1.93; 18.74]
**Total (95% CI)**			**100.0%**	**2.52 [1.21; 5.24]**

Heterogeneity: $Tau^2 = 0.2704$; $Chi^2 = 5.88$, df = 2 (P = 0.05); $I^2 = 66\%$

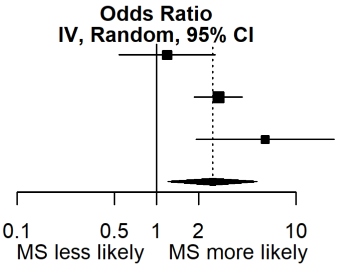

(g)

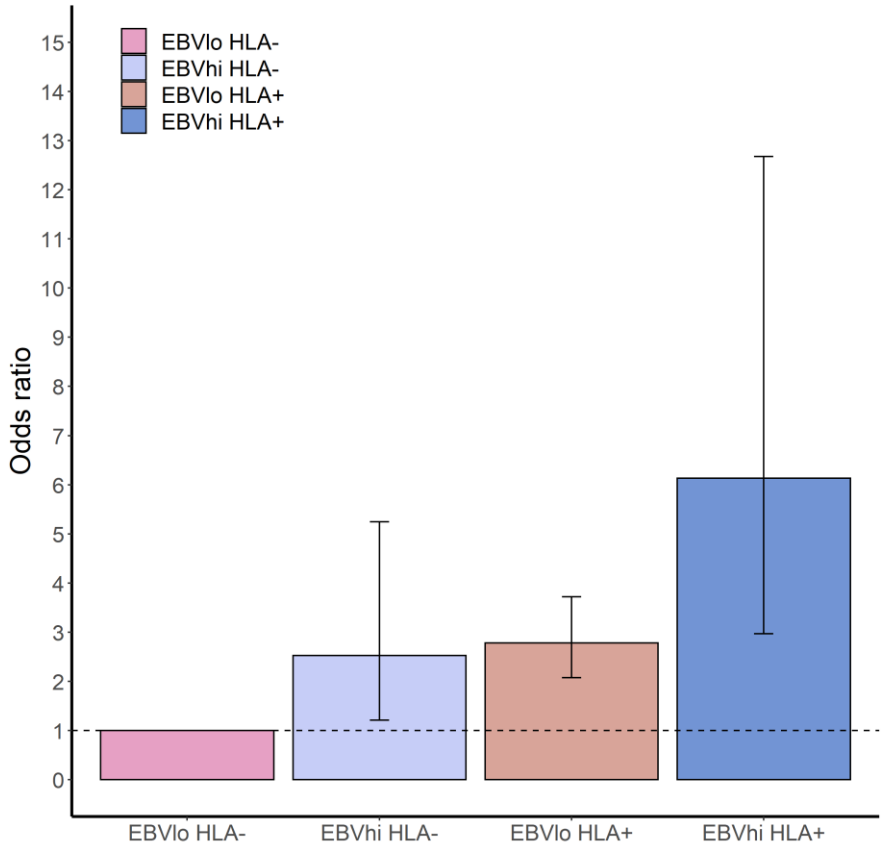

(h)

Supplementary figure 2: (a)-(d) Graphs as per figure 3, analysis restricted to studies using tagging SNPs to determine HLA genotype. (e) - (h) Graphs as per figure 3, analysis restricted to studies using PCR-based methods to determine HLA genotype.

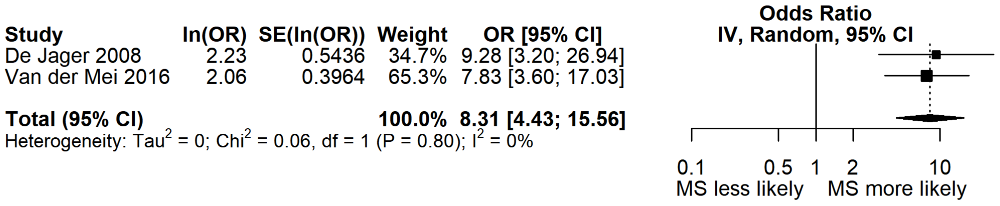

(a)

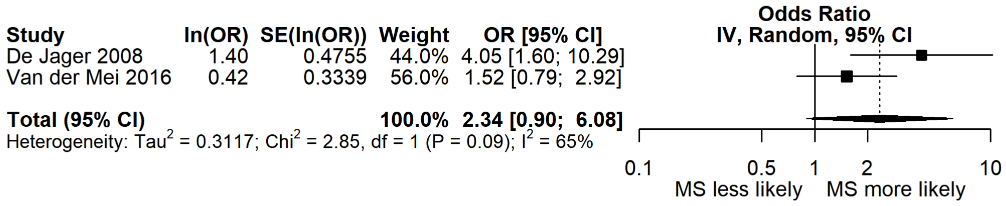

(b)

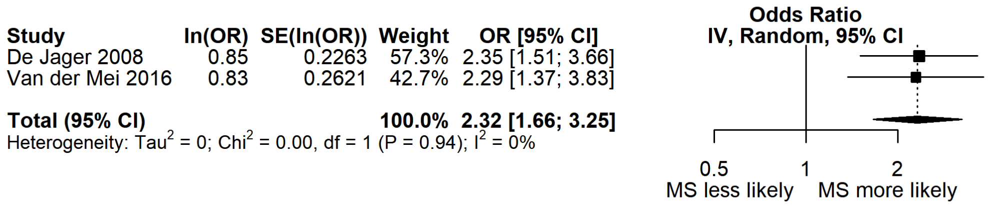

(c)

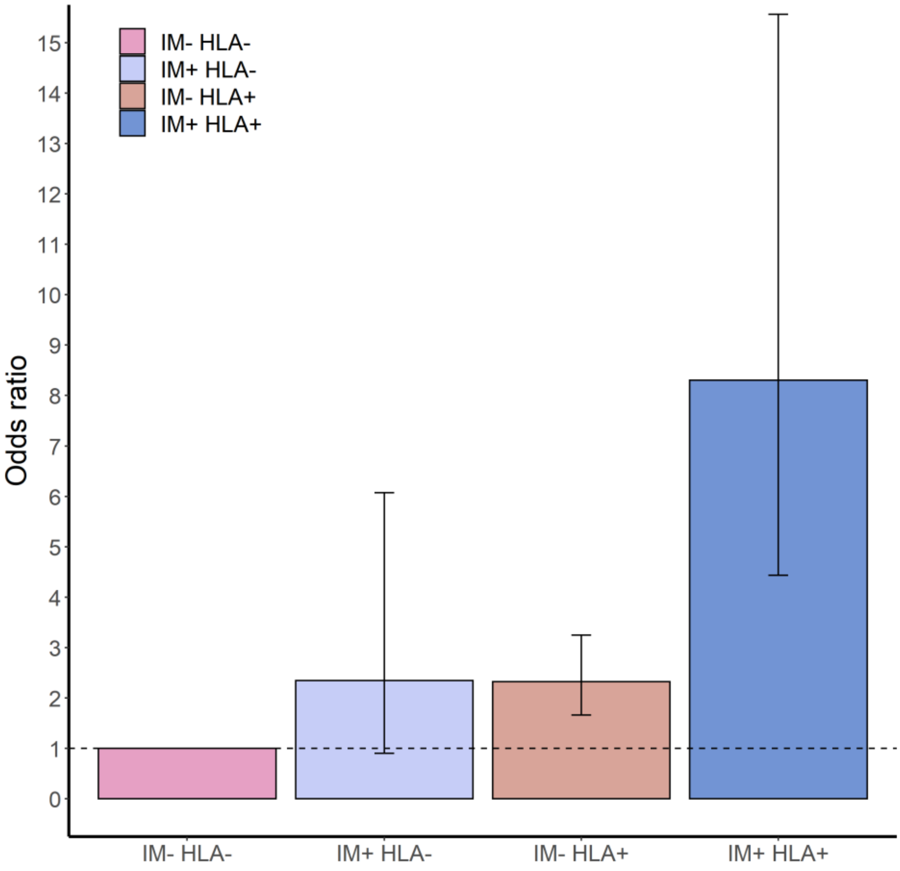

(d)

Study	ln(OR)	SE(ln(OR))	Weight	OR [95% CI]
Nielsen 2009	0.36	0.3088	49.6%	1.43 [0.78; 2.61]
Sundqvist 2011	2.01	0.2695	50.4%	7.49 [4.42; 12.70]
**Total (95% CI)**			**100.0%**	**3.29 [0.65; 16.70]**

Heterogeneity: Tau^2 = 1.2900; Chi^2 = 16.36, df = 1 (P < 0.01); I^2 = 94%

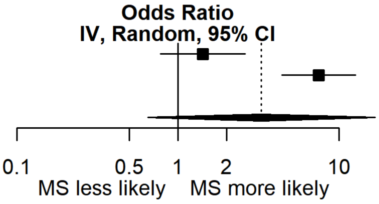

(e)

Study	ln(OR)	SE(ln(OR))	Weight	OR [95% CI]
Nielsen 2009	-1.59	0.2553	49.9%	0.20 [0.12; 0.34]
Sundqvist 2011	0.66	0.2439	50.1%	1.94 [1.20; 3.12]
**Total (95% CI)**			**100.0%**	**0.63 [0.07; 5.71]**

Heterogeneity: Tau^2 = 2.4705; Chi^2 = 40.64, df = 1 (P < 0.01); I^2 = 98%

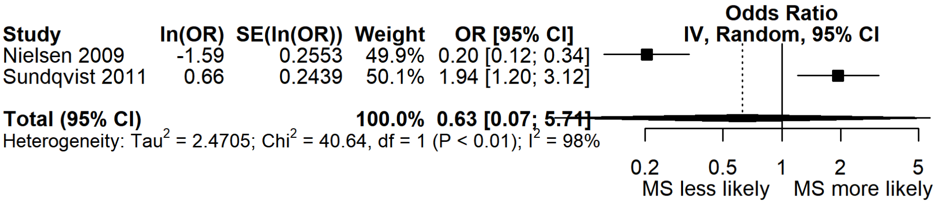

(f)

Study	ln(OR)	SE(ln(OR))	Weight	OR [95% CI]
Nielsen 2009	0.89	0.1069	51.5%	2.43 [1.97; 3.00]
Sundqvist 2011	1.36	0.1348	48.5%	3.88 [2.98; 5.06]
**Total (95% CI)**			**100.0%**	**3.05 [1.93; 4.83]**

Heterogeneity: Tau^2 = 0.0945; Chi^2 = 7.39, df = 1 (P < 0.01); I^2 = 86%

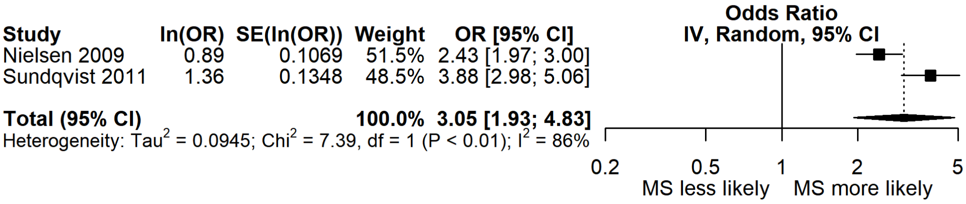

(g)

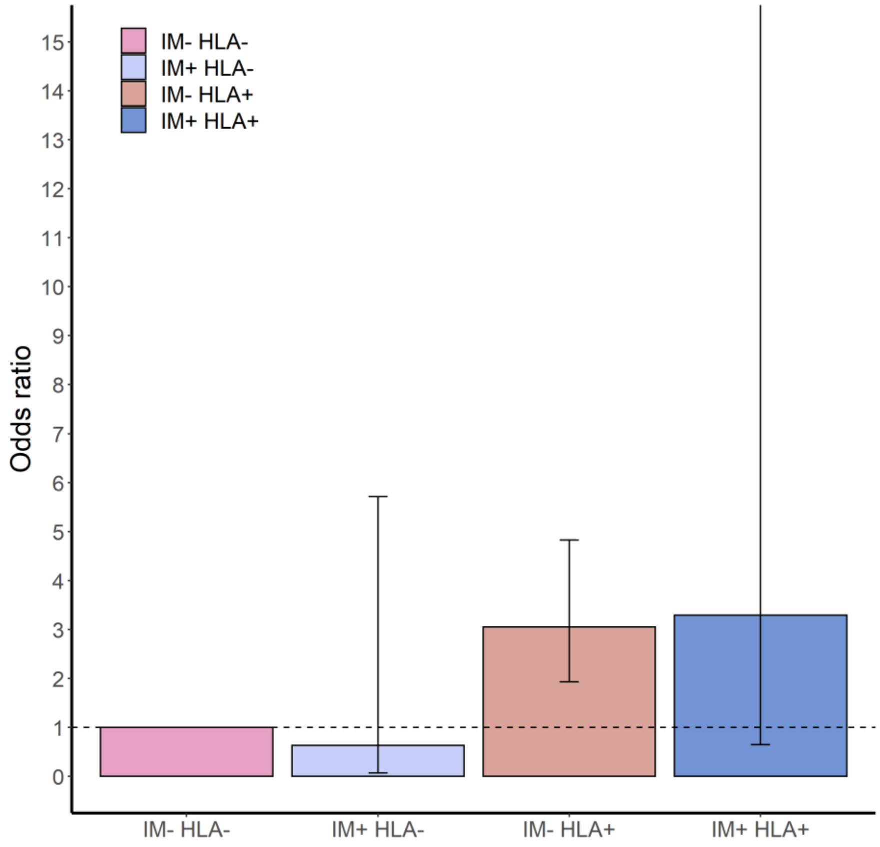

(h)

Supplementary References

References for EBV seropositivity and MS
(i) Adults:
[1][2][3][4][5][6][7][8][9][10][11][12][13][14][15][16][17][18][19][20][21][22][23][24][25][26][27][28][29][30][31][32][33][34][35][36][37][38][39][40][41][42][43][44][45][46][47][48]

(ii) Paediatric MS
[49][50][51][52][53][54][55][56][57][58]

References for IM and MS
[59][60][61][16][28][62][63][64][65][66][67][68][69][70][71][72][73][74][75]

References examining EBV DNA detectable by PCR
[2][76][25][37][44][77][78][79][80][81][82][83][84][85][86][87][88][89]

Bibliography

1. Langer-Gould A, Wu J, Lucas R, Smith J, Gonzales E, Amezcua L, et al. Epstein-Barr virus, cytomegalovirus, and multiple sclerosis susceptibility: A multiethnic study. Neurology. 2017;89: 1330–1337.

2. Agostini S, Mancuso R, Guerini FR, D'Alfonso S, Agliardi C, Hernis A, et al. HLA alleles modulate EBV viral load in multiple sclerosis. J Transl Med. 2018;16: 80.

3. Al-Temaimi R, Alroughani R, Jacob S, Al-Mulla F. Gender influence in EBV antibody response in multiple sclerosis patients from Kuwait. J Neuroimmunol. 2015;285: 57–61.

4. Strautins K, Tschochner M, James I, Choo L, Dunn DS, Pedrini M, et al. Combining HLA-DR risk alleles and anti-Epstein-Barr virus antibody profiles to stratify multiple sclerosis risk. Mult Scler. 2014;20: 286–294.

5. Sundqvist E, Sundström P, Lindén M, Hedström AK, Aloisi F, Hillert J, et al. Lack of replication of interaction between EBNA1 IgG and smoking in risk for multiple sclerosis. Neurology. 2012;79: 1363–1368.

6. Mouhieddine TH, Darwish H, Fawaz L, Yamout B, Tamim H, Khoury SJ. Risk factors for multiple sclerosis and associations with anti-EBV antibody titers. Clin Immunol. 2015;158: 59–66.

7. Ascherio A, Munger KL, Lennette ET, Spiegelman D, Hernán MA, Olek MJ, et al. Epstein-Barr virus antibodies and risk of multiple sclerosis: a prospective study. JAMA. 2001;286: 3083–3088.

8. Awwad AM, Hanafi NF, Achmawi GA, Naguib AM. Epstein-Barr Virus Infection in Multiple Sclerosis Patients. Egypt J Immunol. 2017;24: 49–55.

9. Bray PF, Bloomer LC, Salmon VC, Bagley MH, Larsen PD. Epstein-Barr virus infection and antibody synthesis in patients with multiple sclerosis. Arch Neurol. 1983;40: 406–408.

10. Comabella M, Montalban X, Horga A, Messmer B, Kakalacheva K, Strowig T, et al. Antiviral immune response in patients with multiple sclerosis and healthy siblings. Mult Scler. 2010;16: 355–358.

11. Compston DA, Vakarelis BN, Paul E, McDonald WI, Batchelor JR, Mims CA. Viral infection in patients with multiple sclerosis and HLA-DR matched controls. Brain. 1986;109 (Pt 2): 325–344.

12. Csuka D, Simon D, Hóbor R, Uray K, Prohászka Z, Bánlaki Z, et al. Serum concentration of immunoglobulin G-type antibodies against the whole Epstein-Barr nuclear antigen 1 and its aa35-58 or aa398-404 fragments in the sera of patients with systemic lupus erythematosus and multiple sclerosis. Clin Exp Immunol. 2013;171: 255–262.

13. Décard BF, von Ahsen N, Grunwald T, Streit F, Stroet A, Niggemeier P, et al. Low vitamin D and elevated immunoreactivity against Epstein-Barr virus before first clinical manifestation of multiple sclerosis. J Neurol Neurosurg Psychiatry. 2012;83: 1170–1173.

14. Gieß RM, Pfuhl C, Behrens JR, Rasche L, Freitag E, Khalighy N, et al. Epstein-Barr virus antibodies in serum and DNA load in saliva are not associated with radiological or clinical disease activity in patients with early multiple sclerosis. PLoS One. 2017;12: e0175279.

15. Gutiérrez J, Vergara MJ, Guerrero M, Fernández O, Piédrola G, Morales P, et al. Multiple sclerosis and human herpesvirus 6. Infection. 2002;30: 145–149.

16. Haahr S, Plesner AM, Vestergaard BF, Höllsberg P. A role of late Epstein-Barr virus infection in multiple sclerosis. Acta Neurol Scand. 2004;109: 270–275.

17. Honarmand H, Ahmadi Jalali Moghadam M, Hatamian H, Roudbary A. Possible Relations Between Epstein-Barr Virus Past Infection and Classic Multiple Sclerosis in Guilan, Iran. Jundishapur J Microbiol. 2015;8: e15985.

18. Ingram G, Bugert JJ, Loveless S, Robertson NP. Anti-EBNA-1 IgG is not a reliable marker of multiple sclerosis clinical disease activity. Eur J Neurol. 2010;17: 1386–1389.

19. Karampoor S, Zahednasab H, Pirkouh AA, Monavari SHR, Ramagopalan S, Keyvani H. Serostatus of Epstein-Barr virus in Iranian MS patients. Acta Neurol Belg. 2016;116: 43–46.

20. Lalive PH, Häusler MG, Maurey H, Mikaeloff Y, Tardieu M, Wiendl H, et al. Highly reactive anti-myelin oligodendrocyte glycoprotein antibodies differentiate demyelinating diseases from viral encephalitis in children. Mult Scler. 2011;17: 297–302.

21. Larsen PD, Bloomer LC, Bray PF. Epstein-Barr nuclear antigen and viral capsid antigen antibody titers in multiple sclerosis. Neurology. 1985;35: 435–438.

22. Lavery AM, Collins BN, Waldman AT, Hart CN, Bar-Or A, Marrie RA, et al. The contribution of secondhand tobacco smoke exposure to pediatric multiple sclerosis risk. Mult Scler. 2018; 1352458518757089.

23. Levin LI, Munger KL, Rubertone MV, Peck CA, Lennette ET, Spiegelman D, et al. Temporal relationship between elevation of epstein-barr virus antibody titers and initial onset of neurological symptoms in multiple sclerosis. JAMA. 2005;293: 2496–2500.

24. Lindsey JW, Hatfield LM, Vu T. Epstein-Barr virus neutralizing and early antigen antibodies in multiple sclerosis. Eur J Neurol. 2010;17: 1263–1269.

25. Lünemann JD, Tintoré M, Messmer B, Strowig T, Rovira A, Perkal H, et al. Elevated Epstein-Barr virus-encoded nuclear antigen-1 immune responses predict conversion to multiple sclerosis. Ann Neurol. 2010;67: 159–169.

26. Mameli G, Cossu D, Cocco E, Masala S, Frau J, Marrosu MG, et al. EBNA-1 IgG titers in Sardinian multiple sclerosis patients and controls. J Neuroimmunol. 2013;264: 120–122.

27. Mameli G, Cocco E, Frau J, Marrosu MG, Sechi LA. Epstein Barr Virus and Mycobacterium avium subsp. paratuberculosis peptides are recognized in sera and cerebrospinal fluid of MS patients. Sci Rep. 2016;6: 22401.

28. Martyn CN, Cruddas M, Compston DA. Symptomatic Epstein-Barr virus infection and multiple sclerosis. J Neurol Neurosurg Psychiatry. 1993;56: 167–168.

29. Munch M, Riisom K, Christensen T, Møller-Larsen A, Haahr S. The significance of Epstein-Barr virus seropositivity in multiple sclerosis patients? Acta Neurol Scand. 1998;97: 171–174.

30. Munger KL, Levin LI, O'Reilly EJ, Falk KI, Ascherio A. Anti-Epstein-Barr virus antibodies as serological markers of multiple sclerosis: a prospective study among United States military personnel. Mult Scler. 2011;17: 1185–1193.

31. Myhr KM, Riise T, Barrett-Connor E, Myrmel H, Vedeler C, Grønning M, et al. Altered antibody pattern to Epstein-Barr virus but not to other herpesviruses in multiple sclerosis: a population based case-control study from western Norway. J Neurol Neurosurg Psychiatry. 1998;64: 539–542.

32. Nejati A, Shoja Z, Shahmahmoodi S, Tafakhori A, Mollaei-Kandelous Y, Rezaei F, et al. EBV and vitamin D status in relapsing-remitting multiple sclerosis patients with a unique cytokine signature. Med Microbiol Immunol. 2016;205: 143–154.

33. Nociti V, Frisullo G, Marti A, Luigetti M, Iorio R, Patanella AK, et al. Epstein-Barr virus antibodies in serum and cerebrospinal fluid from multiple sclerosis, chronic inflammatory demyelinating polyradiculoneuropathy and amyotrophic lateral sclerosis. J Neuroimmunol. 2010;225: 149–152.

34. Pandit L, Malli C, D'Cunha A, Shetty R, Singhal B. Association of Epstein-Barr virus infection with multiple sclerosis in India. J Neurol Sci. 2013;325: 86–89.

35. Ponsonby A-L, van der Mei I, Dwyer T, Blizzard L, Taylor B, Kemp A, et al. Exposure to infant siblings during early life and risk of multiple sclerosis. JAMA. 2005;293: 463–469.

36. Ramroodi N, Niazi AA, Sanadgol N, Ganjali Z, Sarabandi V. Evaluation of reactive Epstein-Barr Virus (EBV) in Iranian patient with different subtypes of multiple sclerosis (MS). Braz J Infect Dis. 2013;17: 156–163.

37. Riverol M, Sepulcre J, Fernandez-Diez B, Villoslada P, Fernandez-Alonso M, Rubio M, et al. Antibodies against Epstein-Barr virus and herpesvirus type 6 are associated with the early phases of multiple sclerosis. J Neuroimmunol. 2007;192: 184–185.

38. Schlemm L, Giess RM, Rasche L, Pfuhl C, Wakonig K, Behrens JR, et al. Fine specificity of the antibody response to Epstein-Barr nuclear antigen-2 and other Epstein-Barr virus proteins in patients with clinically isolated syndrome: A peptide microarray-based case-control study. J Neuroimmunol. 2016;297: 56–62.

39. Sellner J, Cepok S, Kalluri SR, Nestler A, Kleiter I, Kümpfel T, et al. Aquaporin 4 antibody positive central nervous system autoimmunity and multiple sclerosis are characterized by a distinct profile of antibodies to herpes viruses. Neurochem Int. 2010;57: 662–667.

40. Shirodaria PV, Haire M, Fleming E, Merrett JD, Hawkins SA, Roberts SD. Viral antibody titers. Comparison in patients with multiple sclerosis and rheumatoid arthritis. Arch Neurol. 1987;44: 1237–1241.

41. Sumaya CV, Myers LW, Ellison GW. Epstein-Barr virus antibodies in multiple sclerosis. Arch Neurol. 1980;37: 94–96.

42. Sumaya CV, Myers LW, Ellison GW, Ench Y. Increased prevalence and titer of Epstein-Barr virus antibodies in patients with multiple sclerosis. Ann Neurol. 1985;17: 371–377.

43. Sundström P, Juto P, Wadell G, Hallmans G, Svenningsson A, Nyström L, et al. An altered immune response to Epstein-Barr virus in multiple sclerosis: a prospective study. Neurology. 2004;62: 2277–2282.

44. Villegas E, Santiago O, Carrillo JA, Sorlózano A, Guerrero M, Fernández O, et al. Low intrathecal immune response of anti-EBNA-1 antibodies and EBV DNA from multiple sclerosis patients. Diagn Microbiol Infect Dis. 2011;70: 85–90.

45. Wagner HJ, Hennig H, Jabs WJ, Siekhaus A, Wessel K, Wandinger KP. Altered prevalence and reactivity of anti-Epstein-Barr virus antibodies in patients with multiple sclerosis. Viral Immunol. 2000;13: 497–502.

46. Wandinger K, Jabs W, Siekhaus A, Bubel S, Trillenberg P, Wagner H, et al. Association between clinical disease activity and Epstein-Barr virus reactivation in MS. Neurology. 2000;55: 178–184.

47. Yoshimura S, Isobe N, Yonekawa T, Matsushita T, Masaki K, Sato S, et al. Genetic and infectious profiles of Japanese multiple sclerosis patients. PLoS One. 2012;7: e48592.

48. Zivadinov R, Nasuelli D, Tommasi MA, Serafin M, Bratina A, Ukmar M, et al. Positivity of cytomegalovirus antibodies predicts a better clinical and radiological outcome in multiple sclerosis patients. Neurol Res. 2006;28: 262–269.

49. Alotaibi S, Kennedy J, Tellier R, Stephens D, Banwell B. Epstein-Barr virus in pediatric multiple sclerosis. JAMA. 2004;291: 1875–1879.

50. Banwell B, Krupp L, Kennedy J, Tellier R, Tenembaum S, Ness J, et al. Clinical features and viral serologies in children with multiple sclerosis: a multinational observational study. Lancet Neurol. 2007;6: 773–781.

51. Banwell B, Bar-Or A, Arnold DL, Sadovnick D, Narayanan S, McGowan M, et al. Clinical, environmental, and genetic determinants of multiple sclerosis in children with acute demyelination: a prospective national cohort study. Lancet Neurol. 2011;10: 436–445.

52. Lünemann JD, Huppke P, Roberts S, Brück W, Gärtner J, Münz C. Broadened and elevated humoral immune response to EBNA1 in pediatric multiple sclerosis. Neurology. 2008;71: 1033–1035.

53. Makhani N, Banwell B, Tellier R, Yea C, McGovern S, O’Mahony J, et al. Viral exposures and MS outcome in a prospective cohort of children with acquired demyelination. Mult Scler. 2016;22: 385–388.

54. Mowry EM, James JA, Krupp LB, Waubant E. Vitamin D status and antibody levels to common viruses in pediatric-onset multiple sclerosis. Mult Scler. 2011;17: 666–671.

55. Pohl D, Krone B, Rostasy K, Kahler E, Brunner E, Lehnert M, et al. High seroprevalence of Epstein-Barr virus in children with multiple sclerosis. Neurology. 2006;67: 2063–2065.

56. Selter RC, Brilot F, Grummel V, Kraus V, Cepok S, Dale RC, et al. Antibody responses to EBV and native MOG in pediatric inflammatory demyelinating CNS diseases. Neurology. 2010;74: 1711–1715.

57. Waubant E, Mowry EM, Krupp L, Chitnis T, Yeh EA, Kuntz N, et al. Common viruses associated with lower pediatric multiple sclerosis risk. Neurology. 2011;76: 1989–1995.

58. Yea C, Tellier R, Chong P, Westmacott G, Marrie RA, Bar-Or A, et al. Epstein-Barr virus in oral shedding of children with multiple sclerosis. Neurology. 2013;81: 1392–1399.

59. Sundqvist E, Sundström P, Lindén M, Hedström AK, Aloisi F, Hillert J, et al. Epstein-Barr virus and multiple sclerosis: interaction with HLA. Genes Immun. 2012;13: 14–20.

60. Gustavsen MW, Page CM, Moen SM, Bjølgerud A, Berg-Hansen P, Nygaard GO, et al. Environmental exposures and the risk of multiple sclerosis investigated in a Norwegian case-control study. BMC Neurol. 2014;14: 196.

61. Bjørnevik K, Riise T, Bostrom I, Casetta I, Cortese M, Granieri E, et al. Negative interaction between smoking and EBV in the risk of multiple sclerosis: The EnvIMS study. Mult Scler. 2017;23: 1018–1024.

62. Claire Simon K, Schmidt H, Loud S, Ascherio A. Epstein-Barr virus candidate genes and multiple sclerosis. Mult Scler Relat Disord. 2015;4: 60–64.

63. Gusev E, Boiko A, Lauer K, Riise T, Deomina T. Environmental risk factors in MS: a case-control study in Moscow. Acta Neurol Scand. 1996;94: 386–394.

64. Haahr S, Koch-Henriksen N, Møller-Larsen A, Eriksen LS, Andersen HM. Increased risk of multiple sclerosis after late Epstein-Barr virus infection: a historical prospective study. Mult Scler. 1995;1: 73–77.

65. Hedström AK, Lima Bomfim I, Hillert J, Olsson T, Alfredsson L. Obesity interacts with infectious mononucleosis in risk of multiple sclerosis. Eur J Neurol. 2015;22: 578–e38.

66. Hernán MA, Zhang SM, Lipworth L, Olek MJ, Ascherio A. Multiple sclerosis and age at infection with common viruses. Epidemiology. 2001;12: 301–306.

67. Lossius A, Riise T, Pugliatti M, Bjørnevik K, Casetta I, Drulovic J, et al. Season of infectious mononucleosis and risk of multiple sclerosis at different latitudes; the EnvIMS Study. Mult Scler. 2014;20: 669–674.

68. Marrie RA, Wolfson C, Sturkenboom MC, Gout O, Heinzlef O, Roullet E, et al. Multiple sclerosis and antecedent infections: a case-control study. Neurology. 2000;54: 2307–2310.

69. Nielsen TR, Rostgaard K, Nielsen NM, Koch-Henriksen N, Haahr S, Sørensen PS, et al. Multiple sclerosis after infectious mononucleosis. Arch Neurol. 2007;64: 72–75.

70. Operskalski EA, Visscher BR, Malmgren RM, Detels R. A case-control study of multiple sclerosis. Neurology. 1989;39: 825–829.

71. Ramagopalan SV, Valdar W, Dyment DA, DeLuca GC, Yee IM, Giovannoni G, et al. Association of infectious mononucleosis with multiple sclerosis. A population-based study. Neuroepidemiology. 2009;32: 257–262.

72. Souberbielle BE, Martin-Mondiere C, O'Brien ME, Carydakis C, Cesaro P, Degos JD. A case-control epidemiological study of MS in the Paris area with particular reference to past disease history and profession. Acta Neurol Scand. 1990;82: 303–310.

73. van der Mei I, Lucas RM, Taylor BV, Valery PC, Dwyer T, Kilpatrick TJ, et al. Population attributable fractions and joint effects of key risk factors for multiple sclerosis. Mult Scler. 2016;22: 461–469.

74. Zaadstra BM, Chorus AMJ, van Buuren S, Kalsbeek H, van Noort JM. Selective association of multiple sclerosis with infectious mononucleosis. Mult Scler. 2008;14: 307–313.

75. Zorzon M, Zivadinov R, Nasuelli D, Dolfini P, Bosco A, Bratina A, et al. Risk factors of multiple sclerosis: a case-control study. Neurol Sci. 2003;24: 242–247.

76. Lucas RM, Ponsonby A-L, Dear K, Valery P, Pender MP, Burrows JM, et al. Current and past Epstein-Barr virus infection in risk of initial CNS demyelination. Neurology. 2011;77: 371–379.

77. Mancuso R, Delbue S, Borghi E, Pagani E, Calvo MG, Caputo D, et al. Increased prevalence of varicella zoster virus DNA in cerebrospinal fluid from patients with multiple sclerosis. J Med Virol. 2007;79: 192–199.

78. Mechelli R, Vittori D, Coarelli G, Aimati L, De Luca O, Romano S, et al. Screening for neurotropic viruses in cerebrospinal fluid of patients with multiple sclerosis and other neurological diseases. Mult Scler. 2014;20: 638.

79. Martin C, Enbom M, Söderström M, Fredrikson S, Dahl H, Lycke J, et al. Absence of seven human herpesviruses, including HHV-6, by polymerase chain reaction in CSF and blood from patients with multiple sclerosis and optic neuritis. Acta Neurol Scand. 1997;95: 280–283.

80. Alvarez-Lafuente R, García-Montojo M, De Las Heras V, Domínguez-Mozo MI, Bartolome M, Benito-Martin MS, et al. Herpesviruses and human endogenous retroviral sequences in the cerebrospinal fluid of multiple sclerosis patients. Mult Scler. 2008;14: 595–601.

81. Morré SA, van Beek J, De Groot CJ, Killestein J, Meijer CJ, Polman CH, et al. Is Epstein-Barr virus present in the CNS of patients with MS? Neurology. 2001;56: 692.

82. Cocuzza CE, Piazza F, Musumeci R, Oggioni D, Andreoni S, Gardinetti M, et al. Quantitative detection of epstein-barr virus DNA in cerebrospinal fluid and blood samples of patients with relapsing-remitting multiple sclerosis. PLoS One. 2014;9: e94497.

83. Ben Fredj N, Rotola A, Nefzi F, Chebel S, Rizzo R, Caselli E, et al. Identification of human herpesviruses 1 to 8 in Tunisian multiple sclerosis patients and healthy blood donors. J Neurovirol. 2012;18: 12–19.

84. Cossu D, Masala S, Cocco E, Paccagnini D, Frau J, Marrosu MG, et al. Are Mycobacterium avium subsp. paratuberculosis and Epstein-Barr virus triggers of multiple sclerosis in Sardinia? Mult Scler. 2012;18: 1181–1184.

85. Hay KA, Tenser RB. Leukotropic herpesviruses in multiple sclerosis. Mult Scler. 2000;6: 66–68.

86. Wagner H-J, Munger KL, Ascherio A. Plasma viral load of Epstein-Barr virus and risk of multiple sclerosis. Eur J Neurol. 2004;11: 833–834.

87. Leibovitch EC, Lin C-TM, Billioux BJ, Graves J, Waubant E, Jacobson S. Prevalence of salivary human herpesviruses in pediatric multiple sclerosis cases and controls. Mult Scler. 2018; 1352458518765654.

88. Santón A, Cristóbal E, Aparicio M, Royuela A, Villar LM, Alvarez-Cermeño JC. High frequency of co-infection by Epstein-Barr virus types 1 and 2 in patients with multiple sclerosis. Mult Scler. 2011;17: 1295–1300.

89. Sotelo J, Ordoñez G, Pineda B. Varicella-zoster virus at relapses of multiple sclerosis. J Neurol. 2007;254: 493–500.
